# Informational Resilience in the Human Cognitive Ecology

**DOI:** 10.3390/e25091247

**Published:** 2023-08-22

**Authors:** Rasmus Gahrn-Andersen

**Affiliations:** Department of Culture and Language, University of Southern Denmark, 4200 Slagelse, Denmark; rga@sdu.dk

**Keywords:** autopoiesis, biosphere, Shannon, enactivism, cognitive science, sociosphere

## Abstract

Resilience is a basic trait of cognitive systems and fundamentally connected to their autopoietic organization. It plays a vital role in maintaining the identity of cognitive systems in the face of external threats and perturbances. However, when examining resilience in the context of autopoiesis, an overlooked issue arises: the autopoietic theory formulated by Maturana and Varela (1980) renders traditional Shannon information obsolete, highlighting that information should not be ascribed a role in cognitive systems in a general sense. This paper examines the current situation and suggests a possible way forward by exploring an affordance-based view on information, derived from radical cognitive science, which is exempted from Maturana and Varela’s critique. Specifically, it argues that the impact of social influence on affordance use is crucial when considering how resilience can manifest in informational relations pertaining to the human cognitive ecology.

## 1. Introduction

The term resilience has gained prominence across various fields, including ecology [1], sociology [2], psychology and the material sciences (for a review, see [3]; the term traces its origins back to the Latin words *resilientem* and *resilire*, which mean ‘inclined to leap, spring or bounce back’ (cf. [3]). This characterization reflects a system’s ability to recover from an external disturbance and return to a stable state. Bunnell [4] observes that the ecological sciences’ understanding of resilience in the 1960s aligned closely with its Latin definition, referring to a system’s ability to stabilize itself after an external disturbance and highlighting its recovery capacity [4] (p. 673). However, resilience also encompasses a slightly different meaning. According to Holling [5], resilience can be defined as “a measure of the persistence of systems and of their ability to absorb change and disturbance and still maintain the same relationships between populations or state variables” [5] (p. 14). This definition implies that the system does not necessarily enter an undesirable state. Instead, resilience becomes synonymous with the system’s basic ability to withstand external shocks or pressures while successfully maintaining its homeostasis. Accordingly, a living system can exhibit resilience in at least two ways: First, there is the system’s general ability to maintain the relations between its state variables amidst the exposure to external perturbations. Such resilience links with the basic fact that the system is a self-reproducing system and, hence, capable of upholding its identity and internal coherence in the face of disruptions. In this first sense, the external perturbances are no serious threat to the system and its resilience is basically identical to the system’s ability to uphold its homeostatic organization. Second, a system can also exhibit resilience in circumstances where its homeostasis is disrupted and where the system would have to undergo various stress reactions before it can return to a state that either resembles its initial (so-called ‘naive’) state or an improved homeostatic state. As explained by Smirnova and colleagues [6], the difference between these states can be cashed out as follows: in the former, the system enters a recovery phase, which leads to its healing with some of the system’s components remaining in their altered states with so-called ‘molecular scars’. In the latter case, there is no need for a recovery given that the system comes to develop robustness or tolerance towards the threat (p. 249).

As introduced by Maturana and Varela [7], the concept of ‘autopoiesis’ seems indispensable when exploring both kinds of resilience in living systems. In their view, a living system is necessarily a cognitive system whose capability for self-reproduction reflects its basic ability to survive through adaptive responses to environmental perturbances even in cases where its homeostasis is disrupted (p. 13). As Beer [8] points out, it does not matter if the internal configuration of the system changes as long as the system survives (p. 66). Following Maturana and Varela [7], living systems do not exist over against their surroundings but are relationally constituted with the environment through a process known as ‘structural coupling’. As Mingers points out, it is the system itself which decides how, when and in relation to what it reacts: “The environmental perturbation can be said only to trigger or select a change of state, not to determine it. Indeed, it is the [system’s] structure itself which determines what can and what cannot be a trigger” [9] (p. 320). Yet, exploring resilience in relation to autopoietic systems comes with a hitherto unexplored consequence: it renders information useless or so it seems. The reason for this is that Maturana and Varela [7] make the provocative claim that the concept of ‘information’ has no applicability in the context of autopoiesis. They write: “Notions such as coding and transmission of information do not enter in the realization of a concrete autopoietic system because they do not refer to actual processes in it” (p. 90). Considering the important role of information in fields including the cognitive sciences [10,11] and semantic biology [12], it begs the question of whether informational relations can be said to play a role in autopoiesis-based resilience.

The present paper explores the possibility of developing a notion of information that accords with autopoietic theory and that allows for investigating resilience in the context of the human cognitive ecology. The paper begins, in Section 2, by nuancing Maturana and Varela’s (1980) dismissal of the concept of information, showing that their criticism has restricted validity in that it is aimed at how information was envisaged by Shannon. Then, in Section 3, I stress how Kauffman and Roli [13] have been making good progress in reconstruing information to make it a better fit with autopoietic theory. Yet, I argue, their affordance-based take on information must be developed to become a better fit with autopoietic theory and to accommodate the role of social influences. Finally, Section 4 explores how information connects with factors pertaining to aspects of the human sociosphere and allows for a particular kind of informational resilience in the human cognitive ecology linked to affordance use in tightly coupled social settings. 

## 2. Shannon Information Vis-À-Vis Autopoietic Theory and Enactivism

I initially stressed that Maturana and Varela [7] consider codes and information as being incompatible with autopoietic theory. However, for the sake of clarity, it is worth exploring their criticism in detail. This will allow us to clarify that their concern is primarily focused on a specific interpretation of information. Specifically, they aim to challenge the ontological foundations of ‘information’ as the term is commonly defined in information theory research, cognitive science and other disciplines. In connection with their thematic exposition on the topic of reproduction in autopoiesis, Maturana and Varela unfold this critique as follows:

“Notions such as coding, a message or information are not applicable to the phenomenon of self-reproduction; their use […] apply only to the reduction of uncertainties in the communicative interactions between independent unities under conditions in which the messenger acts as an arbitrary non-participant link. [I]n self-reproduction there is no transmission of information between independent entities; the reproducing and the reproduced unities are topologically independent entities produced through a single process of autopoiesis in which all components have a constitutive participation” [7] (p. 102).

In this specific context, the concept of information is considered in a manner that closely aligns with Shannon’s framework. This can be observed through the following two points:

First, the terms ‘codes’, ‘message’ and ‘information’ are all central to Shannon’s account. Shannon formulated his *A Mathematical Theory of Communication* in 1948 by examining information in relation to general communication systems and he subsequently developed the theory in collaboration with Weaver in 1949 (see [14]). In this connection, communication is understood as a linear sequential process that unfolds in the following way: an information source generates a message, which is then transmitted (sometimes following an encoding) to a receiver (which may involve decoding), until the message ultimately reaches its intended destination (referred to as “the person (or thing) for whom the message is intended”) (cf. [14], pp. 33–34). 

Second, Maturana and Varela’s criticism targets a particular ontology that is characterized by being both non-relational (or essentialist) and mentalist. As I argue below, this point is particularly crucial since it allows for a way of bringing information into play in the context of autopoiesis presupposed that information is construed in relational and non-mentalist terms. Looking specifically at communication in engineered systems, Shannon and Weaver highlight the distinction between, on the one hand, information and, on the other, the message that is transferred during the communication process [14] (p. 8). It is asserted that information “relates not so much to what you *do* say, as to what you *could* say” (p. 9). The information is not the message being transmitted but what enables an agent to construct a meaningful message in the first place. In this view, information is relative to an agent considered as an independent entity, and it is contingent upon the agent’s decision-making abilities. As such, the unit of information reflects the “amount of freedom of choice” one has when composing a message.

It is difficult to overlook the strong mentalist overtones of Shannon’s definition: this relates to the fact that ‘choices’ play a crucial role in both an explicit and a tacit manner. According to Shannon, information is the logarithm of the number of choices available when composing a message at a given point in time. Information is entropic in the sense that “it is expressed in terms of the various probabilities involved—those of getting to certain stages in the process of forming messages, and the probabilities that, when in those stages, certain symbols be chosen next” (p. 12). Thus, the stochastic nature of composing a message can occur at various levels, including (1) the choice of symbols within a word; (2) the choice of words and symbols, including punctuation marks like commas and full stops, within a sentence; (3) the construction of a sentence within a paragraph; and so on. In essence, information processing revolves around the basic task of reducing entropy through a series of Markov chains, which can be seen as essentially binary in nature. Krippendorff writes: “While the base of this logarithm is arbitrary, Shannon set it to two, thereby acknowledging that the choice among two equally likely alternatives […] is the most elementary choice conceivable” [15] (p. 614). Indeed, the fundamental unit of information corresponds to an event with a probability of occurrence of ½. This leads us to a more implicit type of choice: the choice that marks the end of the stochastic chain involved in composing the message. In written text, this can be illustrated by the use of a final ‘full stop’, which concludes the last sentence and, consequently, the text as a whole. However, the specific semantic marker for concluding a message may vary across different communication media. For example, in radio communication (e.g., walkie-talkies), the customary use of “over” signifies that the message has been completed, whereas in letter writing, the sender typically includes a signature as the concluding element. It is the information source who ultimately decides when and how to bring the decision-making process to a close, thereby eliminating any further options for choice in the composition of the message. Hence, when it comes to the message, a different type of choice is required to eliminate the remaining residue of entropy. While Shannon and Weaver push the view that “the entropy (or information) associated with the process which generates messages or signals is determined by the statistical character of the process” [14] (p. 17), the statistical nature alone is insufficient for ultimately determining informational outcomes. They require another kind of choice at a different epistemic level, specifically the level pertaining to the transmission process as a whole, which effectively enables the finalization of the message composition (for an example of a binary theory in a unitary form, see Spencer-Brown’s *Laws of Form* (cf. [16], p. 9)). 

Although Shannon and Weaver refrain from evoking cognitivist concepts such as ‘mind’, ‘consciousness’ and ‘brain’, their account does carry certain mentalist undertones due to how information is being construed as relative to ‘choices’ or ‘decisions’. This gives the clear impression that information processing takes place within the realm of the brain or, at the very least, is driven by mind-internal computational processes similar to those later explored by mentalist (or representationalist) cognitive science (e.g., [10]). This connection is closely related to the criticism put forth by Maturana and Varela. Information thus construed is tied to what they term as ‘heteropoiesis’, which pertains to the realm of human (artificial) design, not living beings [7] (p. 90). So, rather than pertaining to cognitive systems as such, heteropoiesis is the outcome or product of specific cognitive dynamics and, hence, should not be confused with them. As such, the phenomenality of heteropoietic systems is a domain of description imposed by the human observer who specifies elements, relations and sequential orders amongst different kinds of phenomena. As the quote above suggests, this is different from the thoroughly interconnected internal complexity of living, autopoietic systems. 

Thus, it is in this light that we should understand Maturana and Varela’s criticism. Their criticism pertains to how information is construed in semantical biology, cognitive science and other fields where Shannon’s original conception of information is dominating. In the context of cognitive science, however, it is worth mentioning that proponents of so-called radical or enactivist positions are equally critical of standard mentalist models and Shannon information. As Barandiaran [17] notes, enactivism as it is introduced by Varela et al. [18] rejects “the linear sequence ‘Sense (input) → Plan (compute) → Action (output)’ where cognition stands on the ‘planning side’, often under the form of symbolic propositional (or otherwise content driven) computational processing” [17] (p. 410). Going back to Shannon’s model, we see how such a view resonates with how he breaks up communication into a linear process where the generation of a message (or ‘computation’) and the transmission of the message (‘action’) are construed as separate stages in a linear process. Considered in relation to cognition, the view corresponds to mentalist models that take information processing to enable action (see [10]). On an enactivist view, however, action and thinking cannot be separated. Varela famously exemplified this with his programmatic claim that cognition is analogous to ‘laying down a path while walking’. Relatedly, Hutto and Myin [19] argue that “the embedded and embodied activity of living beings provides the right model for understanding minds” and, thus, that in order “[t]o understand mentality, however complex and sophisticated it may be, it is necessary to appreciate how living beings dynamically interact with their environments” (p. 4). Accordingly, Hutto and Myin stress the fact that so-called ‘basic minds’ are not only free of informational content but also extensive in the sense that they are relationally constituted while engaging with their surroundings. In this connection, Hutto and Myin make the provocative claim that informational content does not exist in nature as such but is rather a product of human language use and particular human-specific practices, which have given rise to propositions with truth content [19] (p. xv). 

## 3. Beyond Heteropoiesis: Affordance-Based Organism–Environment Relations as Constitutive of Information

Although Maturana and Varela introduce autopoiesis while critiquing Shannon information, we find that autopoietic theory has the potential for being related to information construed somewhat differently. In fact, Maturana and Varela’s criticism should not make us avoid using the concept of information but rather force us to clarify how the notion can be developed in a relational fashion so that it does not contrast with the non-heteropoietic core of autopoietic theory. What I aim to show in this section is that we can make a move towards a relationalist and non-essentialist conception of information grounded in human affordance use.

Relativism is central to autopoietic theory. This is evident from how Maturana and Varela criticize Shannon information for presupposing the existence of ‘independent entities’ (e.g., coding, a message and information). In Maturana and Varela’s view, such entities only exist relative to a description made by a human observer. For this reason, there can be no talk about the transmission of information (in Shannon’s sense) between living entities that do not co-embody or share a particular descriptive domain (cf. the quote above by Maturana and Varela). To put it differently, notions articulated in the so-called “language of heteropoietic design” are descriptions of living processes made by an observer but not themselves fully representative of the processes they describe. This is because they denote entities as separate from one another rather than as being functionally intertwined. Kauffman and Roli [13] push an autopoiesis-based account informed by insights from ecological psychology and, in particular, the Gibsonian notion of affordances. Effectively, they make the case for considering external artefacts and tools—or simply affordances—as having a constitutive role in the autopoietic operations of organisms. Affordances are relational in the sense that “an affordance is an entangled property of both the organism and its environment” (p. 2). In terms of definition, Kauffman and Roli define an affordance as “‘The use of *X* to accomplish *Y*’, where *X* may be, e.g., an object or a living being (as an affordance can be a feature of another organism), and *Y* is in general an action, a behavior, or a biological function” [13] (p. 2). They argue that what Gibson [20] conceptualizes as ‘affordances’ should be central to not just the actions of agents but even constitutive, at least in part, of the agents and their environments. Accordingly, affordances allow for the ‘becoming of the biosphere’. In this connection, Kauffman and Roli build on Gibson’s ecological psychology to make the case that mathematical set theory can only be used to model evolutionary traits in organisms in a synchronic manner but not diachronically. The reason for this is that, in being relationally constituted, an affordance does not have a definite number of functions or usages. Instead, they can be used in many ways, meaning a multitude of future uses are possible:

“How many uses of a screwdriver alone or with other things are there? Is the number of uses a specific number? Seventeen? Two hundred and thirteen? No. Is the number infinite? How would we know? The universe really is non-ergodic. We could not have used a screwdriver to short an electric connection in the year 1265 A.D. We can do so now. The number of uses of a screwdriver alone or with other things is indefinite. We have no idea now what new uses of a screwdriver may turn up in the next 1000 years” [13] (p. 4).

What is crucial in this regard is the basic fact that affordances are not restricted to strictly habitual uses and, hence, predictable behavioral outcomes. They can also give rise to ‘novel adaptations’, which come in at least two forms: either as new solutions to existing problems or as new tools. In both instances, however, they come to play a vital part in how organisms self-construct in relation to their environment. In fact, in their account, Kauffman and Roli downplay recurrency in the use of affordances in favor of a focus on the ever-evolving usage potential of such affordances. It is in relation to such novel adaptations that new information is created. Kauffman and Roli write the following:

“We suggest that the ever new-in-the-universe possibilities that emerge as organisms construct themselves and get to exist for some time constitute the creation of new information. It is this propagating construction of new possibilities that is seized and breathes fire into the evolving biosphere” [13] (p. 5).

By acknowledging that affordances can instigate informational relations, Kauffman and Roli identify a potentially fruitful basis for an alternative to Shannon information in the context of autopoietic theory. Specifically, they argue that, in contrast to Shannon information, “which propagates already existing syntactic information” [21] (p. 7) that stands over against semantic meaning, we must recognize that affordances “are carved out of a continuum, e.g., bumps and dents become new relevant variables in a formal representation of the world. After the relevant variables have co-created themselves, we ‘can name them’” (ibid.). The fit between affordances and autopoiesis is further exemplified by their claim that affordances cannot be defined in a non-circular way in the sense that they form part of both the organism and its environment in the outset [13] (p. 2 and 7). This also resonates with how Maturana and Varela [7] define autopoiesis where an organismic autopoietic self is itself a prerequisite for—as well as a result of—autopoietic processes, thus making it prudent to consider the so-called ‘circularity of self-reference’ (pp. 78–79). 

Despite this obvious overall fit with autopoietic theory, it can nevertheless be argued that Kauffman and Roli’s take on information is not radical enough in the context of enactivist cognitive science and, moreover, that there is at least one indication of the fact that it might actually be at odds with autopoietic theory. The reason for this is that Kauffman and Roli take information to be semantical:

“In the evolution of the biosphere, *semantics*, the functional use of *X* to do *Y*, *precedes any syntactic symbols* we may later use in a mathematical model. In short, “possible uses of X” are affordances seized by heritable variation and natural selection and *become semantic adaptive features* of evolving Kantian Wholes…” [13] (p. 5).

By ascribing a key role to semantics and, hence, informational content, Kauffman and Roli mirror the so-called ‘received view’, which is dominant in traditional cognitive science where semantic information is taken to be synonymous with mental content [19] (p. 37). This is unfortunate given how it brings heteropoiesis back into the game in the sense that mentalists qua the received view tend to assume that cognizers stand over against the world. Indeed, as Maturana and Varela explain, we should be careful in not confusing cognitive terms that describe events in an observed domain with the processes that constitute the domain [7] (p. 90). By embracing informational content, Kauffman and Roli treat information as semantic meaning and, hence, as something that, analytically at least, can be distinguished from what carries it. For as pointed out by Hutto and Myin, informational content relies on, to the very least, an analytical content-vehicle distinction. This distinction entails that content can be seen in separation from whatever ‘carries’ or ‘contains’ it and, further, as something that is “somehow distinct from the way an organism responds to the worldly offerings in intentionally directed and perhaps emotionally charged ways” [19] (p. 35). The vehicle in Kauffman and Roli’s account is the affordance. Consequently, it can be argued that Kauffman and Roli end up embracing heteropoiesis, which contrasts with their self-declared commitment to autopoiesis (cf. Maturana and Varela’s criticism of heteropoiesis). Despite Roli and Kauffman’s initial recognition of the fact that “affordances are carved out of a continuum” [21] (p. 7), the presence of the content-vehicle dualism suggests that distinct processes are in play—that information can somehow be seen apart from what instantiates it.

However, the good news is that it does not need to be so in the sense that Gibson’s take on information is ambiguous As shown by van Dijk and Kiverstein [22], Gibson [20] operates with two conflicting senses in which a medium can grant agents so-called ‘direct perception’: one sense is the so-called ‘ready-made view’ where the medium and the information available to the cognizer are taken to exist prior to an act of perception. For an example of such a view, see de Carvalho and Rolla [23], who consider ecological information as information *for* action and, in so doing, embrace the idea that “stimulus information is structured energy to which an organism may be sensitive” (for another proponent of this view, see [24] (pp. 106 and 109)). It appears to be in this particular sense that Kauffman and Roli consider affordance-based informational relations despite also mentioning that affordance-based information is relationally constituted. This is evident from how their account relies on an analytical distinction between medium and information or simply: vehicle and content. This distinction relates to the assumption that information can exist prior to being perceived and that it allows for being transmitted, produced and consumed. Yet, there are also examples of Gibson considering the medium as what van Dijk and Kiverstein [22] term ‘usage-based information’. In contrast to the former, this kind of affordance-related information is not pre-existing in the environment, rather it comes to be relationally constituted as an organism engages with its surroundings through acts of direct perception. Specifically, “it is by actively establishing the relation to its surrounds that the organism perceives what the environment affords” [22] (p. 8391). In contrast to the former, this view takes information to be co-constituted with affordance use and, hence, as a relational and non-heteropoietic phenomenon. Van Dijk and Kiverstein’s take on affordances can inform us to avoid recourse to heteropoiesis. In this view, information should not be seen in separation from the affordance that manifests it. Instead, information is intrinsic to the functionality of affordances and, as such, indistinguishable from how they are actually used. Put differently, it is only as it is being put to use that an affordance can constitute an informational relation between the organism and its environment. Importantly, this does not lead to heteropoiesis given that it avoids falling into the trap of considering information as something that exists apart from the interacting organism or the affordances used (cf. Maturana and Varela’s critique of the language of heteropoietic design). In being relationally constituted, affordances are here defined in basic accordance with the biosemiotic take on affordances that Kauffman and Roli adopt, testifying to the fact that “an affordance is an entangled property of both the organism and its environment” [13] (p. 2—my underlining) but without presupposing the workings of informational content. 

Having thus argued that an appeal to affordance-relative information need not entail semantic information, there is another reason for bringing certain nuances to Kauffman and Roli’s account. This is underlined by the fact that it is not seemingly possible to locate resilience in relation to information structures. For in the view of Kauffman and Roli, affordances have an indefinite number of potential applications, thus underlining that they may change—or evolve—based on how they shape the ecosphere. The fact that affordances are not immune to the ‘arrow of time’ (cf. [25]) is of course related to the fact that natural processes are irreversible and, hence, that living systems evolve. So, the question remains if it is at all possible to trace resilience to the information connected to an organism’s use of affordances. In the next section, I critically engage with Kauffman and Roli’s overemphasis on non-ergodicity, arguing that there are also key traits characteristic of human affordance use that exhibit stability or reoccurrence over time and, in particular, that it is in relation to these that informational resilience exhibits itself and shows to be of crucial importance to the functional upholding of the human sociosphere.

## 4. Resilience in Affordance-Based Information

In the context of human cognition, it is important to bear in mind that the human cognitive ecology does not involve just a biosphere but also a sociosphere or simply: culture. To paraphrase Hutchins, our brains have co-evolved with cultural norms and values. Yet, traditional cognitive science has so far not been interested in exploring their interdependencies. As Hutchins puts it,

“One of the biggest challenges of the coming decades will be working out the implications of the fact that for humans, the ‘world’ (in the now familiar ‘brain–body–world’ formulation) consists of culturally constructed social and material settings. The advent of culture is, after all, the transformative event in the history of the human mind” [26] (p. 711).

It would be naïve to think that affordances can somehow be exempted from social influences and, further, abstracted away from their specific contexts of practical applicability. Human beings do not just appropriate naturally occurring objects as affordances for our cognition. We also design, produce and reproduce them and we do so in accordance with particular standards and rules, and with the purpose of fulfilling certain purposes, in accordance with social norms, values, etc. (see, e.g., [27]). Thus, the socio-material aspects of our cognitive ecology are shaped by and shaping of the affordances we use including tools, instruments, artefacts, totems, maps, etc. In fact, even words—which indicate the hallmark of our “intelligence” and what many take to be what truly distinguishes us from other species—may be said to have tool-like qualities and, hence, be affordance-like (cf. [28]). 

Rietveld and Kiverstein emphasize the point that affordances cannot be seen in isolation from the social practices that condition their usage and, hence, co-determine their functionality. Specifically, they hold that affordances “are dependent on the abilities available in a particular ecological niche” [29] (p. 326) and, consequently, on social practices. This point corresponds with Heidegger’s [30] phenomenology, which recognizes that useful things are functionally determinate in the sense of having their ‘in-order-to’ [Umzu], which, amongst other things, involves a reference to other useful things that condition their use. Most importantly, how we use a given thing or tool “subordinates itself to” the predetermination that follows from our practical world where different useful things exist side by side [30] (p. 69). Thus, as Heidegger shows, there is a pre-extant organization of things to our cognitive ecology, which allows for a useful thing to appear as such in the first place [30] (p. 68). In this connection, it is worth noting Heidegger’s point concerning that there is “no such as a thing as *a* useful thing”. Rather, useful things always belong to “a totality of useful things in which this useful thing can be what it is” (ibid.). In-order-to’s do vary across practices and change over time, which is also what Kauffman and Roli’s screwdriver example suggests. Nevertheless, we should keep in mind that our affordance use cannot be abstracted away from its actual context of practical application in a sociosphere and, hence, the myriad of other useful things, or affordances, that determine their use. The point here is to avoid falling into the trap of focusing merely on the potential usage of an affordance as do Kauffman and Roli but to adopt a sociospheric focus by exploring the usages that are permitted and constrained by certain standards and values and, more specifically, a practice which self-sustains or self-reproduces by means of recurrent norms and rules that unfold on much slower time scales than physical phenomena (cf. [31]). Also, we must bear in mind that human agents normally have acquired a sufficient degree of expertise through training or experience to be able to perform simple and complex tasks in particular practical settings. As such, the use of basic tools for problem solving is part of this. Learning undertaken by a biological organism in a constant environment, as construed by Zhang [32], is characterized by the fact that entropy is reduced to zero in that “having completed its learning, [the agent] has no uncertainty about the best way to respond to this environment” [33] (p. 14). Seen in relation to competent affordance use, this is no exception: Heidegger [30] shows that proficient tool use entails a high level of certainty that the agent focuses, not on the tool itself, but on the task at hand. Thus, the tool becomes an embodied extension of the agent and is experientially ignored as a thing-in-itself. Relatedly, in the context of ecological psychology and ecological enactivism, it is often pointed out that affordances are ‘nested’. Their nested nature testifies to at least two crucial aspects pertaining to affordance use in human social practices. First, that affordances can exist within affordances and, hence, be compounded as exemplified by technically sophisticated and multipurposed affordances such as computers, cars and space rockets. Second, that affordances also ‘comprise of multiple sequential actions’ [34], meaning that their use is closely connected with behaviors that unfold sequentially as part of practical task performance. This second point underpins the strongly regulated and rule-based nature of human affordance use and, further, the fact that practical activities and their related tasks bear on other activities and tasks and, as such, are predictable to the extent that they do not face contingencies. This is particularly salient in the case of those human social practices that are tightly coupled in the sense that there is a closed, rule-bound logic that determines how sequences of actions relate to one another in the context of a given practice. In being tightly coupled, such practices are synonymous with “tightly-constrained activity domains, composed of well-structured problems and highly organised infrastructures” [35] (p. 147). In his influential 1995 book, *Cognition in the Wild*, Hutchins [36] studies how cognition is distributed on the USS Palau and provides numerous examples of the tightly structured work practices onboard the vessel. For our current purposes, it makes sense to consider one example in particular: an incident where USS Palau had suffered a loss of power and was facing a possible collision with a sailboat. Normally, the vessel would have alarmed any approaching boat that a collision was imminent, using its steam whistle to sound five blasts as a warning. Yet, due to a lack of steam pressure, this was not possible. So, the crew had to resort to a manual foghorn instead. This involved a crew member having to sprint across the deck in order to sound the much less powerful foghorn as close as possible to the approaching vessel [36] (pp. 1–6). What this highly condensed summary of Hutchins example shows is that it is not only the material traits of affordances that are important to their functioning in social practices but, equally so, their functional traits and, further, how they connect with other elements of a practice.

Although Kauffman and Roli, in their accounts [13,21], predominantly abstain from explicitly thematizing the role of entropy in the context of affordances, we must recognize that entropy plays a role. Friston [37] argues that there is a clear connection between the autopoietic organization of biological systems and how these systems are characterized by their resistance to the second law of thermodynamics. Accordingly, living systems aim towards maintaining their homeostasis by means of entropy reduction. As Gallagher points out, this links to the fact that an “unbounded increase of entropy [is equal to] systemic death” [38] (p. 7). In the context of Friston’s work on predictive brains, the reduction in entropy entails that an organism minimizes its free energy in the form of its own prediction errors, thus aiming to eliminate the possibility of ‘surprises’. Surprises come about due to an incongruence between the organism’s beliefs or expectations and the actual states it embodies. While Friston treats biological self-organization without considering the role of affordances, Kauffman and Roli’s account puts affordances in the mix, thus recognizing that, at least in the case of humans, our cognitive ecology is bound to unfold through the use of tools and other artefacts. Thus, affordances are considered as crucial mediators between us as organisms and the niche we exist in (see also [27,39]). In this connection; however, it should be noted that Roli and Kauffman [21] exemplify a limited take on affordance use in the sense that they take affordances to emerge from “bumps and dents”, which the organism encounters in the world, thus ignoring the fact that affordances are also used or encountered in the absence of entropy or surprises (see also [40]). As long as they are functionally similar, affordances can easily substitute each other, thus allowing for a system to uphold—or, in the case of USS Palau, to rebalance—its homeostasis in the face of external threats and perturbances. Thus, “bumps and dents” (to paraphrase Roli and Kauffman) may not necessarily give rise to new affordances. Rather, the increase in systemic disorder—or entropy—resulting from such perturbing events can instead be mitigated by means of recurrency in affordance-based informational relations. The functional substitution of affordances effectively reduced entropy in the distributed cognitive system of the USS Palau to such an extent that it enabled the two vessels to systemic co-function, thus avoiding a maritime disaster. Both the foghorn and the steam whistle can be used to sound collision warnings. What the foghorn shares with the whistle is an invariant ‘in-order-to’, thus giving evidence of a high degree of predictability in terms of its use and the linked behavioral outcomes. Historically, in maritime practices, even cannons have been serving the same function. Although having different material configurations, it can be argued that cannons, whistles and foghorns are informationally invariant in the sense that as affordances belonging to a particular maritime practice, they can reify functionally identical informational relations. In situations where two ships are on a collision course, all of these three affordances can effectively reduce prediction errors and limit systemic entropy in the navigation practice. This tells us at least two things: First, in acknowledging that affordances pertain not just to a biosphere but also a sociosphere, affordances do indeed have definite and recurrent usages. They are localized in time and space in certain practical contexts where they functionally relate to the performance of predefined and recurrent tasks. This point is crucial when considering how human social practices can reproduce and uphold themselves amid perturbances and threats. Second, they testify to the fact that information relations exhibit resilience in the sense that they are enacted over and over again. Both points tie to the recursive nature of the human sociosphere and culture more generally which, as Luhmann [41] shows, have clear autopoietic traits (as to how recursion fits autopoietic theory, see [42]), thus testifying to the, in part at least, predictable nature of human affordance usage in the context of well-established, tightly coupled social practices. In relation to informational resilience, it can thus be argued that it is the nested nature of affordances and the fact that they are conditioned and constrained by stable cultural patterns that allow for a sufficient degree of predictability in a physical world that, considered in its own right, is non-ergodic and characterized by irreversible flux and transformation.

## 5. Conclusions

Having evolved across various disciplines, the term ‘resilience’ denotes a system’s capacity to withstand or recover from external perturbances. Resilience is linked to the system’s basic ability to adapt and survive in response to changes in the environment. Autopoietic systems are cognitive systems fundamentally characterized by their ability to withstand pressure from their surroundings. Resilience thus connects with the basic fact that autopoietic systems are operationally closed and self-reproducing, meaning that they engage with the world on the basis of their own internally generated norms and values. Yet, considering that Maturana and Varela introduce autopoiesis by criticizing the so-called ‘heteropoietic nature’ of Shannon information, it remains unclear if an alternative conception of information is possible in the context of autopoietic theory and, above all, how such a conception can be related to the resilience of autopoietic systems in the human cognitive ecology. Indeed, the realm of heteropoiesis differs fundamentally from that of autopoiesis in that it characterizes, not the living as such, but the artificial and, hence, the humanly designed. So, although heteropoietic phenomena are constituted through cognitive processes, such phenomena should not be confused with the basic, autopoietic or cognitive processes that allow for their emergence. In the view of Maturana and Varela, basic cognition is autopoietic, not heteropoietic. Having highlighted the incongruency between autopoietic theory (as developed by Maturana and Varela) and Shannon-style information, the paper went on to identify a promising informational alternative in the form of Kauffman and Roli’s affordance-based approach that, in the outset at least, seems aligned with not just autopoietic theory but also radical, anti-representationalist positions in cognitive science. Thus, on the basis of their account, it seems possible to develop a relationalist notion of information, which differs from its non-relationalist (or essentialist) counterpart as conceived by Shannon. Kauffman and Roli make the sensible move of considering organisms as being fundamentally related to their surroundings by means of affordance use. Thus, it is the affordance-based relations themselves that are informational. However, there is a two-fold downside: First, that Kauffman and Roli fail to adequately distinguish their notion of information from the one introduced by Shannon given how they safeguard an appeal to ‘semantic meaning’. Second, that their focus on the biosphere leaves the crucial influences of the sociosphere out of the picture and, critically, makes it difficult to consider the information involved in affordance use as exhibitory of resilience. My claim is that both problems can be avoided by considering how the sociosphere influences affordance use. Thus, I make the case that human cognition is intricately intertwined with cultural norms, values and practices. Affordances, whether naturally occurring or designed by humans, are shaped and influenced by social factors and remain subject to specific standards, rules and purposes determined by social communities and practices. The use of affordances is closely connected to sequential actions within task performance and highlights the rule-based nature of human affordance use. While the material traits of affordances are important including how their use influences the biosphere, their functional traits and how they connect with other elements of a particular sociosphere are equally significant. Thus, they cannot be isolated from the social practices that condition their use, and the nested nature of affordances underscores their dependence on stable cultural patterns. The informational relations intrinsic to affordance use exhibit resilience as they have the potential for being repeatedly enacted in the context of recurrent tasks. This is underlined by the fact that affordances can be functionally substituted as long as they serve a similar purpose. As such, they are localized in specific contexts and are enacted within stable cultural patterns, providing a degree of predictability in a dynamic and ever-changing physical world. Overall, understanding the interplay between affordances and socio-cultural factors is crucial for comprehending the complexity of human cognition and its relationship with the external environment and, on theoretical grounds, for showing that autopoietic theory can acknowledge the existence of informational relations. 

## Data Availability

Not applicable.
